# Insulin Monotherapy for Hypertriglyceridemic Pancreatitis in Non-diabetic Patients: A Case Report and Literature Review

**DOI:** 10.7759/cureus.83057

**Published:** 2025-04-27

**Authors:** Maitha A Alshamsi, Maitha T Al Teneiji, Raghavendra Bhat

**Affiliations:** 1 Internal Medicine, RAK Medical and Health Sciences University, Ras Al Khaimah, ARE

**Keywords:** acute pancreatitis, hypertriglyceridemia, hypertriglyceridemic pancreatitis, insulin therapy, non-diabetics, triglyceride

## Abstract

Hypertriglyceridemic pancreatitis (HTGP) is a severe disease that is associated with a higher complication rate compared to pancreatitis caused by other etiologies, and its severity increases in conjunction with triglyceride levels. We report a case of a 57-year-old male with a history of well-controlled hypertension and chronic smoking who presented to the emergency department with clinical, biochemical, and radiological findings suggestive of acute pancreatitis. He had no history of alcohol consumption, and abdominal ultrasound showed no evidence of gallstones, which ruled out both alcoholic and biliary pancreatitis. His lipemic blood specimens and highly elevated serum triglyceride level of 1818 mg/dL confirmed hypertriglyceridemia-induced pancreatic damage. He was managed with conventional acute pancreatitis treatment, including intravenous hydration, bowel rest, and pain medications. Additionally, he was started on an insulin infusion at 0.1 units/kg/hour combined with 10% dextrose to maintain euglycemia. Remarkably, his triglyceride levels dropped significantly over four days to below 500 mg/dL. The patient was discharged in stable condition on anti-hyperlipidemic medication to prevent further attacks. This case emphasizes the importance of recognizing hypertriglyceridemia as an etiological factor of pancreatitis. It also demonstrates the safety and effectiveness of intravenous (IV) insulin infusion in rapidly reducing triglyceride levels in non-diabetic patients. We discuss the proposed pathophysiology, risk factors, and different treatment modalities used for the management of hypertriglyceridemia-induced pancreatitis.

## Introduction

Acute pancreatitis (AP) is among the most prevalent gastrointestinal conditions resulting in hospital admissions. While the majority of cases are primarily attributed to gallstones and alcohol consumption, hypertriglyceridemia (HTG) is considered the third most common cause of AP, accounting for 4-10 % of severe AP admissions worldwide [1,2]. Hypertriglyceridemic pancreatitis (HTGP) occurs when excess chylomicrons are hydrolyzed by pancreatic lipase, releasing free fatty acids (FFAs) that cause microvascular injury and acidosis, precipitating pancreatic inflammation. HTG is defined as fasting triglyceride levels ≥ 150 mg/dL, with severe HTG defined as levels ≥1,000 mg/dL. The threshold level of triglycerides that may trigger pancreatitis varies among individuals. While levels above 1,000 mg/dL are thought to be necessary to initiate the inflammatory process in the pancreas, some literature reports cases occurring at levels closer to 500 mg/dL [3-5]. 

Although HTGP has a clinical presentation similar to AP of other etiologies, its clinical course is more severe. Therefore, identifying HTG as the cause of AP is essential because there are specific therapeutic options unique to HTGP [6]. HTGP should be suspected in patients with risk factors of HTG, which include uncontrolled diabetes, familial hypertriglyceridemia, and obesity [7]. Management of the acute phase of HTGP mandates a comprehensive approach targeting both AP and underlying hypertriglyceridemia [8]. Long-term management focuses on lifestyle modifications, dietary adjustments, and pharmacological therapies to prevent recurrence and maintain serum triglyceride levels within a safe range [9]. Evidence for insulin-only regimens in non-diabetic HTGP is limited to small series; optimal dosing and safety monitoring protocols remain undefined.

## Case presentation

A 57-year-old obese male presented to the emergency department with a history of severe, continuous, and progressively worsening abdominal pain over the past two days. The pain started suddenly in the left upper quadrant of the abdomen and subsequently spread throughout the left side of the abdomen, chest, and shoulder. The pain was associated with fever and bloating. The patient denied any vomiting, diarrhea, black stools, weight loss, or night sweats. His past medical history is significant for hypertension, which is well-controlled by 5 mg perindopril. He reported smoking one pack of cigarettes per day for more than 20 years and denied alcohol consumption. The rest of the history, including surgical and family history, was unremarkable. Also, there was no previous episode of pancreatitis. His temperature was 38.6 ℃, respiratory rate was 16/min, pulse was 119/ min, and blood pressure was 127/82 mmHg. On physical examination, abdominal palpation revealed generalized tenderness. No ecchymotic discoloration, rebound tenderness, hepatosplenomegaly, palpable masses, or abnormal bowel sounds were detected. The rest of the physical examination was unremarkable. 

**Figure 1:**
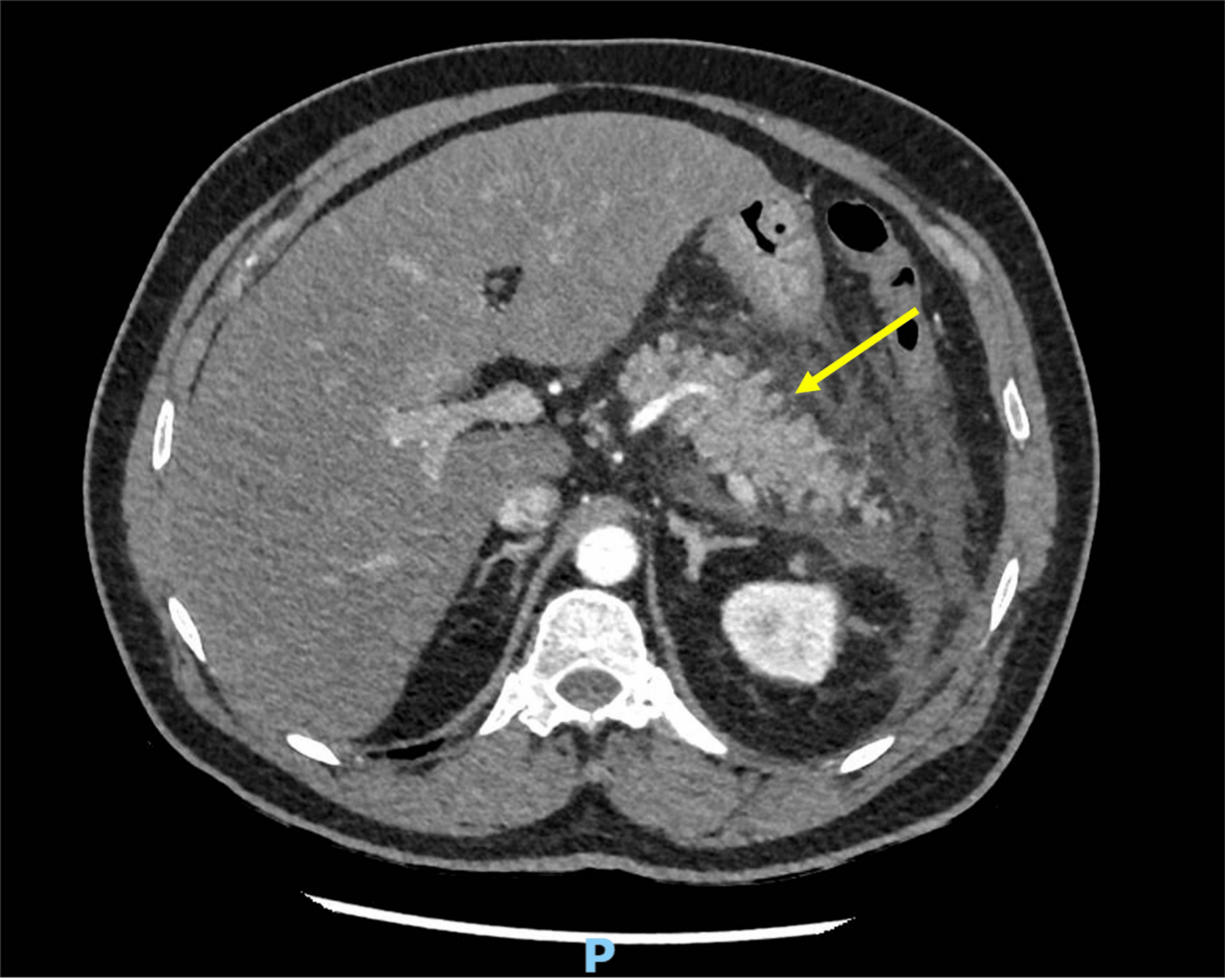
Abdominal CT scan axial view showing changes suggestive of acute pancreatitis (arrow) The Image shows bulky pancreatic body and tail with peripancreatic inflammatory changes.

Initial blood samples revealed highly lipemic specimens. Laboratory tests showed a serum triglyceride level of 1818 mg/dL, serum lipase level of 1356 U/L, and amylase level of 238 U/L. Electrolyte levels included sodium at 121 mmol/L, potassium at 5.7 mmol/L, and calcium at 2.3 mmol/L. Renal function tests showed a creatinine level of 59 μmol/L, blood urea nitrogen (BUN) of 3.1, and an estimated glomerular filtration rate (eGFR) of 123 mL/min/1.73 m² (Table 1). An abdominal ultrasonography was performed, which ruled out cholelithiasis. A computed tomography (CT) scan of the abdomen revealed homogeneous enhancement with a bulky pancreatic body and tail, peripancreatic inflammatory changes evident by peripancreatic fat stranding, and sheets of fluid density with extension of the inflammatory changes to the anterior and lateral left paranephric spaces, and inferiorly to the level of the left iliac fossa. There was no evidence of pancreatic or peripancreatic fat necrosis, drainable collection, or vascular complications (Figure 1). 

**Table 1:** Laboratory investigations Highly elevated serum amylase and lipase, along with elevated triglycerides, suggestive of hypertriglyceridemic induced pancreatitis.

Laboratory Test	Result	Reference Range
White Blood Cell (WBC)	14.61 × 10^3^/mcL	4.5-11 ×10^3^/mcL
Red Blood Cell (RBC)	5.24 ×10^6^/μL	4.2-6×10^6^/μL
Hemoglobin (Hgb)	14.4 g/dL	13-18 g/dL
Hematocrit (Hct)	49.3%	42-52 %
Mean Corpuscular Volume (MCV)	88.3 fL	80-100 fL
Mean Corpuscular Hemoglobin (MCH)	27.2 pg	27-32 pg
Mean Corpuscular Hemoglobin Concentration (MCHC)	33.6 gm/dl	32-36 g/dL
Red cell Distribution Width (RDW)	14.4 %	12-15 %
Platelet Count	277 × 10^3^ /mcL	150-450 ×10^3^/mcL
Mean Platelet Volume (MPV)	11.1 fL	6 - 12 fL
Neutrophil %	63 %	40 - 60 %
Lymphocyte %	46 %	20 - 40 %
Monocyte %	7.4 %	2 - 8 %
Eosinophil %	3 %	1 - 4 %
Basophil %	0.2 %	0.5 - 1 %
C-Reactive Protein (CRP)	57.5 mg/dL	< 0.3 mg/dL
Amylase	238 U/L	38 - 149 U/L
Lipase	1356 U/L	8 - 55 U/L
Triglycerides	1818 mg/dL	< 150 mg/dL
Cholesterol	390 mg/dL	< 200 mg/dL
Sodium	121 mmol/L	135 - 145 mmol/L
Potassium	5.7 mmol/L	3.5 - 5 mmol/L
Calcium	2.3 mmol/L	2.2 - 2.7 mmol/L
Aspartate Aminotransferase (AST)	67 U/L	8 - 33 U/L
Alanine Aminotransferase (ALT)	31 U/L	7 - 56 U/L
Alkaline Phosphatase (ALP)	76 U/L	44 - 147 U/L
Direct Bilirubin	4 µmol/L	< 5.1 µmol/L
Creatinine	59 µmol/L	53 - 106 µmol/L
Blood Urea Nitrogen (BUN)	3.1 mmol/L	2.1 - 8.5 mmol/L
Estimated Glomerular Filtration Rate (eGFR)	123 mL/min/1.73 m^2^	> 90 mL/min/1.73 m^2^

Based on the clinical, laboratory, and radiological findings, a diagnosis of acute pancreatitis secondary to hypertriglyceridemia was made. The patient was kept nil by mouth, hydrated with intravenous fluids, and received pain medications. An insulin infusion was started at a rate of 0.1 units/kg/hour along with 10% dextrose. This approach was selected primarily due to cost considerations, as plasmapheresis was not a financially feasible option for our patient. Blood sugar levels were monitored hourly to avoid hypoglycemia. Adjustments were made to the dextrose infusion rate, and the insulin dosage was titrated accordingly to maintain safe glucose levels. The patient responded well to the intravenous insulin treatment, and his triglyceride level markedly improved, with no episodes of hypoglycemia or other adverse effects observed during treatment (Table 2). Subsequently, he was started on a liquid diet, which was gradually progressed to a regular diet. The patient was discharged in stable condition, and a daily regimen of 40 mg rosuvastatin was initiated to optimize his lipid profile. No follow-up was done for the patient; therefore, no data is available on long-term triglyceride levels or recurrence of pancreatitis.

**Table 2:** Trend of serum triglyceride levels during the first 5 days of hospitalization Triglyceride levels dropped to below 500 mg/dL within four days of treatment.

Day	Day 1	Day 2	Day 3	Day 4	Day 5
Triglycerides (mg/dL)	1818	521	514	461	443

## Discussion

Hypertriglyceridemia (HTG) and hypercalcemia are important metabolic causes that can independently precipitate AP. HTG is the third leading cause of AP, causing between 4% and 10% of episodes. These episodes can be severe and life-threatening, especially with triglyceride levels exceeding 2,000 mg/dL, which are associated with a 10-20% increased risk of pancreatitis [9]. The mechanism by which elevated triglyceride levels cause AP is not fully understood. One leading hypothesis suggests that triglyceride-rich lipoproteins at high levels interact with pancreatic lipase in the pancreatic capillaries, breaking down triglycerides into free fatty acids (FFAs) and lysophosphatidylcholine. These breakdown products damage platelets, vascular endothelium, and acinar cells, causing ischemia and acidosis. Acidosis further increases FFA toxicity by activating trypsinogen, thereby triggering AP. Additionally, elevated chylomicron levels increase plasma viscosity, leading to stasis and hypoxia. This also triggers acidosis and eventually stimulates AP. Both mechanisms likely contribute to the development of HTGP [3,9]. 

HTGP usually occurs in individuals with HTG combined with secondary factors like poorly controlled diabetes, alcoholism, pregnancy, medications, or genetic abnormalities. Genetic studies have found mutations in the cystic fibrosis transmembrane conductance regulator (CFTR) and tumor necrosis factor (TNF) promoter that may play a role in HTGP in some patients, particularly in recurrent or early-onset cases. Large cohort analyses have shown that mutations in the CFTR gene, commonly associated with cystic fibrosis, are present in approximately 6-8% of patients with idiopathic pancreatitis, implicating impaired ductal function and increased susceptibility to pancreatic injury [10]. Additionally, polymorphisms in the promoter region of the TNF gene, particularly TNF-α-308G>A, have been linked to increased expression of pro-inflammatory cytokines, potentially exacerbating the severity and recurrence of HTGP [11]. 

Early management of acute pancreatitis and the prevention of its complications are crucial. Initial conservative treatment should begin promptly upon suspected diagnosis and includes aggressive intravenous hydration, initial bowel rest, and pain control. Several targeted treatment modalities for HTGP have been described, including insulin, heparin, plasmapheresis, combined blood purification therapy (CBPT), high-volume hemofiltration (HVHF), and hemoperfusion (HP) [5,12]. 

Insulin infusion has been used to reduce triglyceride levels for over a decade, effectively lowering levels by 50-75% over two to three days. It can be used for both diabetic and non-diabetic patients. However, frequent monitoring of blood glucose levels is necessary to prevent hypoglycemia [13]. Intravenous (IV) insulin is more effective than subcutaneous insulin, with the standard approach being 0.1-0.3 units/kg/h IV regular insulin along with dextrose saline to maintain blood glucose between 150 and 200 mg/dL [14]. Insulin lowers triglyceride levels by enhancing lipoprotein lipase (LPL) and inhibiting hormone-sensitive lipase (HSL) in adipocytes [14,15]. LPL catalyses the hydrolysis of triglycerides in chylomicrons and very-low-density lipoproteins into glycerol and fatty acids, promoting their storage in adipocytes. HSL in adipose tissue reduces the lipolysis of endogenous triglycerides and subsequent release of FFAs into circulation [16].

Plasmapheresis quickly removes triglycerides and chylomicrons from the circulation, eliminating the inciting factor and halting further pancreatic inflammation and damage. It is reported to reduce triglyceride levels by 50-80% per session. Heparin, when used in combination with insulin, releases stored LPL to reduce triglyceride levels but carries risks such as rebound hypertriglyceridemia and hemorrhage with continuous use. Thus, long-term use of heparin in HTG-induced AP is not recommended [17,18]. 

We conducted a literature search using the PubMed database based on clearly defined inclusion and exclusion criteria. Studies were included if they met the following conditions: (1) involved adult patients (> 18 years) diagnosed with HTGP with serum triglyceride levels exceeding 1000 mg/dL, (2) focused on insulin therapy and its efficacy in reducing triglyceride levels, (3) original studies, including randomized controlled trials, cohort studies, case series, or case reports, (4) were published between the year 2000 and April 2025 to ensure relevance to current clinical practice, and (5) were published in English. Studies were excluded if they met any of the following criteria: (1) involved patients with pre-existing diabetes mellitus, pregnant women, or individuals under 18 years of age, (2) were non-clinical publications such as editorials, commentaries, or non-peer-reviewed articles, or (3) included co-interventions such as plasmapheresis or therapeutic anticoagulation. Based on these criteria, our search using the PubMed database yielded a total of four retrospective studies [19-22] and nine case reports [8,23-30] included in this review.

In the review of retrospective studies, no study was found to have been conducted exclusively on non-diabetic patients with HTGP. However, relevant data were extracted specifically for subgroups without diabetes within broader study populations (Table 3). Tabone et al. reported a 71.87% reduction in triglyceride levels after 48 hours of insulin infusion, a decline comparable to that observed in plasmapheresis-treated patients [19]. Yu et al. demonstrated significantly fewer therapy-related complications in both intensive and non-intensive insulin therapy groups compared to plasma exchange, with no notable differences in clinical outcomes such as mortality or cure rate across the groups [20]. Pulipati et al. highlighted that non-diabetic patients achieved triglyceride reduction goals more quickly and with lower insulin requirements than those with diabetes [21]. Similarly, Perez et al. observed that patients without diabetes reached target triglyceride levels significantly faster than those with diabetes, who also presented with higher baseline levels [22].

**Table 3:** Retrospective review of insulin monotherapy in patients with non-diabetic hypertriglyceridemic pancreatitis. CII: continuous insulin infusion, OTT: onset to treatment time, IIT:  intensive insulin therapy, NIIT: non-intensive insulin therapy, PE: Plasma Exchange, TG: Triglyceride, DM: Diabetes Mellitus, BMI: Body Mass Index, HTGP: Hypertriglyceridemic Pancreatitis

Author and publication year	Country	Sample size	Gender (M/F)	Age (years)	Baseline Triglyceride (mg/dL)	Insulin type and dose	Length of stay (days)	Adverse events to treatment	Key finding
Tabone et al., (2020) [19]	Australia	n=1	Female	48	1930	Not mentioned	Three	None	Conservative therapy ± insulin is as effective as plasmapheresis in reducing serum triglycerides.
Yu et al., (2020) [20]	China	n=46 *Received IIT	30 / 16	39.00±11.01	2499.56 (2088.57-3445.53)	Continuous intravenous infusion (0.1-0.3 units/kg/h)	16.50 (12.00-27.0)	Hypokalemia (n=1)	The study demonstrated that the OTT of patients in the IIT and NIIT groups was decreased compared with that in the PE group.
		n=43 *Received NIIT	31 /12	37.49±9.66	2322.41 (1668.73-3582.82)	Continuous intravenous injection of insulin	14.0 (7.0-22.0)	Hypoglycemia (n=2)	
Pulipati et al., (2021) [21]	USA	n=20	13 / 7	41.3 ± 9.0	3426.5 [2186.5, 4519.0]	0.1 units/kg regular insulin bolus	Not mentioned	Not mentioned	The faster response of serum TG to CII was strongly associated with absence of DM, lower BMI, initial TG, and higher daily insulin use per kg body weight.
Perez el at., (2023) [22]	USA	n=13	Not mentioned	Not mentioned	2532 (1562-3943)	Intravenous insulin (0.1 units/kg/h up to a maximum of 15 units/h)	Five (four-six)	None	Patients with concomitant diabetes mellitus and HTG-AP presented with higher initial TG levels and took significantly longer to achieve a TG level ≤1000 mg/dL.

A total of nine case reports were reviewed (Table 4), with patient ages ranging from 23 to 48 years. While diabetes is a well-established risk factor for hypertriglyceridemia, the reviewed cases show that in non-diabetic patients, it can also be triggered by factors such as alcohol consumption, obesity, genetic predisposition, and certain medications [8,26]. Across the nine reports, patients presented with a wide range of triglyceride levels, from moderate to extremely severe elevations exceeding 10,000 mg/dL. Despite the variation in severity, all patients responded well to insulin therapy, with significant reductions in serum triglyceride and complete clinical recovery reported in each case. The majority received intravenous regular insulin at doses of approximately 0.1 units/kg/hour. Notably, adverse events were minimal, with only two cases of hypoglycemia were reported, with no associated morbidity or mortality [8,23-30]. 

**Table 4:** Literature review of insulin monotherapy in patients with non-diabetic hypertriglyceridemic pancreatitis TG: Triglycerides, HTG: Hypertriglyceridemia, HTGP: Hypertriglyceridemic Pancreatitis

Author and publication year	Country	Age / Gender	Cause of hypertriglyceridemia	Baseline Triglyceride (mg/dL)	Insulin type and dose	Time to TG <500 mg/dL (days)	Length of stay (days)	Adverse events to treatment	Outcome	Key finding
Santos et al., (2017) [23]	USA	47/ Male	Not mentioned	3568	Intravenous insulin (0.1 units/kg/h)	Not mentioned	Not mentioned	None	Recovery	After 12 h of intravenous insulin, a reduction in triglycerides was visually apparent.
Inayat et al., (2018) [8]	Pakistan	39 / Male	Not mentioned	5047	Insulin infusion (0.1 units/kg/h)	12	Not mentioned	None	Recovery	The study highlights insulin monotherapy as an effective treatment option with promising outcomes.
Xie et al., (2018) [24]	USA	31 / Male	Not mentioned	3052	Insulin infusion (0.1 units/kg/h)	Not mentioned	Three	Hypoglycemia	Recovery	The case reveals that insulin can be administered safely and efficiently in a non-diabetic HTGP patient.
Gayam et al., (2018) [25]	USA	48 / Male	Not mentioned	10,612	Insulin infusion (1-2 units/kg/day)	7	Not mentioned	None	Recovery	The study emphasized the quick and effective response to insulin therapy alone.
Sahu et al., (2019) [26]	USA	36 / Female	Alcoholism	1870	Intravenous infusion of regular insulin (0.1 units/kg/h)	1	Not mentioned	None	Recovery	Insulin was continued until next 24 hours until triglyceride level came down below 500 mg/dL.
Soliman S, (2021) [27]	USA	38 / Male	Alcoholism and obesity	2,625	Not mentioned	< 2	Not mentioned	None	Recovery	Intensive insulin therapy is not inferior to plasmapheresis and can be an effective, cheaper, and safe therapy.
Reed et al., (2021) [28]	USA	34 / Female	Familial	3496	Intravenous insulin infusion (0.07 units/kg/h)	4	Four	Hypoglycemia	Recovery	A reduced insulin infusion rate of 0.07 unit/kg per hour was effective at acutely lowering triglyceride levels.
Ahern et al., (2022) [29]	USA	23 / Male	Not mentioned	5794	Infusion of regular insulin (0.1 units/kg/h)	4	Not mentioned	None	Recovery	The patient’s TG level was checked every 8 to 12 hours and decreased to 453 mg/dL by the fourth day.
Ashfaq et al., (2023) [30]	USA	43 / Female	Pembrolizumab	2043	Not mentioned	7	Not mentioned	None	Recovery	Pembrolizumab-associated HTG-causing pancreatitis can be successfully managed with insulin therapy to lower serum TG levels.

This review has several limitations. The number of eligible studies is relatively small, particularly regarding non-diabetic patients with HTGP treated with insulin therapy alone, which limits the generalizability of the findings. Additionally, none of the included retrospective studies focused solely on non-diabetic patients; data for this subgroup were extracted from broader populations, which may introduce bias and affect interpretability.

## Conclusions

The key to recovery from acute pancreatitis due to any etiology includes fluid resuscitation, pain management, and bowel rest. These measures halt the progression of the disease and allow the pancreas time to heal. However, pancreatitis secondary to hypertriglyceridemia necessitates additional specific therapies to decrease serum triglyceride levels. To date, there are no established international guidelines for the management of HTGP. Several treatment modalities, mainly including plasmapheresis, insulin, and heparin, have been described in the literature. These therapeutic strategies have been used either in isolation or in various combinations. Intravenous insulin monotherapy appears to be a promising therapeutic approach for HTGP but remains insufficiently studied. Therefore, further studies are needed to establish a solid treatment algorithm for HTGP, especially in non-diabetic patients. Additionally, there is a pressing need for well-designed comparative studies to evaluate the safety, effectiveness, and outcomes of plasmapheresis, heparin, and combination therapies in non-diabetic patients with HTGP in order to guide optimal clinical decision-making.
